# Bidirectional Relationships between Sarcopenia and Pelvic Floor Disorders

**DOI:** 10.3390/ijerph21070879

**Published:** 2024-07-05

**Authors:** Yacov Grosman, Leonid Kalichman

**Affiliations:** 1 Department of Physical Therapy, Recanati School for Community Health Professions, Faculty of Health Sciences, Ben-Gurion University of the Negev, P.O. Box 653, Beer Sheva 84105, Israel; 2 Department of Physical Therapy, Meuhedet Health Maintenance Organization, Hadera 3824242, Israel

**Keywords:** sarcopenia, pelvic floor disorders, bidirectional relationship, shared risk factors, multidisciplinary care

## Abstract

Sarcopenia and pelvic floor disorders (PFDs) are prevalent and often cooccurring conditions in the aging population. However, their bidirectional relationship and underlying mechanisms remain underexplored. This narrative review aims to elucidate this relationship by exploring potential causative interplays, shared pathophysiological mechanisms, and common risk factors. A comprehensive literature search was conducted to identify relevant studies focusing on epidemiological associations, interaction mechanisms, and implications for patient care. While epidemiological studies demonstrate associations between sarcopenia and PFDs, our findings reveal a cyclical relationship where sarcopenia may exacerbate PFDs through mechanisms such as decreased muscle strength and mobility. Conversely, the presence of PFDs often leads to reduced physical activity due to discomfort and mobility issues, which in turn exacerbate the muscle atrophy associated with sarcopenia. Additionally, shared risk factors such as physical inactivity, nutritional deficiencies, metabolic syndrome, and menopausal hormonal changes likely contribute to the onset and progression of both conditions. These interactions underscore the importance of concurrently integrated care approaches that address both conditions. Effective management requires comprehensive screening, the recognition of contributing factors, and tailored exercise regimens supported by a multidisciplinary approach. Future research should focus on longitudinal studies tracking disease progression and evaluating the efficacy of multidisciplinary care models in optimizing patient outcomes.

## 1. Introduction

As global longevity increases, the demographic shift towards an older population introduces significant challenges to healthcare systems worldwide, including managing the higher prevalence of chronic diseases, accommodating the increased demand for geriatric care, and addressing the financial strain on healthcare resources. According to the United Nations’ Department of Economic and Social Affairs, Population Division [1], the proportion of the global population aged 65 and above is projected to rise from 10% in 2022 to 16%, reaching 1.5 billion, by 2050. Simultaneously, the number of adults aged 80 or older is expected to triple between 2020 and 2050. However, extended lifespans do not necessarily equate to extended healthy life years, often being accompanied by disability, an increased risk of chronic diseases, and diminished quality of life [2]. These issues pose a heavy economic and psychosocial burden on patients and significantly strain healthcare system budgets [3]. Among the chronic conditions impacting older adults, sarcopenia and pelvic floor disorders (PFDs) are notable for their profound effects on daily functioning and overall well-being [4,5]. Sarcopenia, characterized by the progressive loss of skeletal muscle mass and function, significantly impacts physical health and mobility, thereby increasing the risk of falls, frailty, and a cascade of related health complications [5]. Concurrently, PFDs, broadly defined as conditions that affect the function and integrity of the pelvic floor muscles, nerves, and connective tissue, encompass a spectrum of issues, including urinary and fecal incontinence (FI) and pelvic organ prolapse (POP) [6,7]. These disorders significantly contribute to morbidity and disability among the elderly, particularly among women, due to factors such as childbirth, hormonal changes during menopause, and anatomical differences [8,9,10]. Beyond their physical impacts, these disorders profoundly affect psychological well-being and social engagement, diminishing overall quality of life [4,11]. Furthermore, emerging evidence suggests that the interplay between sarcopenia and PFDs can exacerbate the severity of both conditions, indicating a complex, bidirectional relationship that amplifies their individual impacts [12].

Despite their significant impacts on the elderly, leading to diminished quality of life and increased healthcare utilization, the intersection of sarcopenia and PFDs remains largely underexplored. This oversight is particularly concerning given that these conditions are often managed within the narrow confines of geriatrics, urogynecology, physical therapy, and potentially other relevant fields, such as nutrition and psychology, leading to compartmentalized care that fails to address the interconnectedness of sarcopenia and PFDs. Such compartmentalization overlooks the potential for these conditions to exacerbate each other and reveals a significant gap in geriatric healthcare, highlighting the urgent need to shift from isolated treatment modalities to a more comprehensive, interdisciplinary approach. Such a shift would not only bridge the existing divide in healthcare practices but also enhance patient outcomes by addressing the bidirectional relationship between muscle strength, pelvic floor integrity, and overall health. 

Currently, there is a lack of comprehensive literature examining the bidirectional relationship between sarcopenia and PFDs. This review addresses this gap by synthesizing existing studies and highlighting the need for integrated research and clinical approaches.

This review aims to explore the interplay between sarcopenia and PFDs, focusing on how these conditions interact and affect each other, emphasizing the implications for patient care and treatment strategies.

## 2. Methods

*Literature Search Strategy*: A comprehensive literature search was performed across PubMed, Scopus, EBSCO, and Google Scholar to identify articles relevant to sarcopenia and PFDs in the context of aging, particularly in older adult women. The search strategy combined keywords and Medical Subject Headings (MeSH) terms, including “sarcopenia”, “pelvic floor disorders”, “urinary incontinence”, “pelvic organ prolapse”, “aging”, “fecal incontinence”, and “women’s health”. Boolean operators and wildcard characters were employed to refine the search further. The following specific research phrases were used: “sarcopenia AND urinary incontinence”, “sarcopenia AND fecal incontinence”, “sarcopenia AND pelvic organ prolapse”, “aging AND sarcopenia”, “aging AND pelvic floor disorders”, “women’s health AND sarcopenia”, and “women’s health AND pelvic floor disorders”. Searches were conducted iteratively, refining the terms based on the initial findings to ensure the inclusion of relevant studies. The search was restricted to articles published in English from inception until January 2024. This time frame was chosen to capture the complete historical development and the most recent advancements in the field, ensuring a comprehensive review. The language restriction to English was applied to ensure the accurate interpretation and analysis of the studies.

*Selection Criteria*: Articles were selected that discussed the epidemiology, pathophysiology, diagnosis, treatment, or management of sarcopenia and PFDs, or their interrelationships, specifically within the aging female population. This encompassed both observational (cross-sectional, cohort, case–control) and interventional (randomized controlled trials, clinical trials) studies. Review articles, meta-analyses, systematic reviews, and guidelines were also included to ensure a broad overview. Studies involving broader demographics were included if they provided relevant insights applicable to older adult women. The exclusion criteria excluded studies that did not provide specific data or insights into the aging female population.

*Data Extraction*: Titles and abstracts were initially screened by two independent reviewers for relevance. The full texts of the selected articles were then reviewed to confirm their inclusion, with any disagreements resolved through consensus. A standardized data extraction form was used to collect information on the study design, participant demographics, interventions or exposures, outcomes measured, and key findings. The extraction form ensured consistent data collection across studies, capturing all relevant information for a comprehensive analysis.

*Data Synthesis*: Data synthesis was conducted narratively, organizing the findings according to the review’s objectives. This included identifying and integrating key themes from the literature to build a comprehensive narrative around the relationship between sarcopenia and PFDs in aging women, their impacts on health and functionality, and their implications for treatment strategies. The narrative synthesis was structured to highlight epidemiological associations, interaction mechanisms, and clinical implications. Criteria such as the study design’s appropriateness, sample size considerations, and methodological clarity in the statistical analysis were rigorously applied to ensure the robustness of the included studies for this narrative review.

## 3. Results and Discussion

### 3.1. Sarcopenia

The term sarcopenia, introduced by Irwin Rosenberg in the late 1980s, translates from Greek to ‘loss of flesh’, underscoring the essence of the condition—age-related muscle deterioration [13]. Its recognition as a distinct disease entity was marked by the allocation of an ICD-10-CM code (M62.84) in 2016 [14]. Sarcopenia has been defined as a progressive and generalized skeletal muscle disease that results in a significant reduction in muscle strength, muscle quantity, or quality. Severe cases are further distinguished by additional deficits in physical performance [14,15]. 

Even in the realm of “healthy aging”, there is a consistent decline in skeletal muscle quality, marked by changes in structure, mechanics, and function [16]. Research indicates that adults lose approximately 25% of their peak muscle mass between the ages of 40 and 70, with this decline accelerating beyond the age of 70 with a gradual decline of about 2% each year [17,18,19]. Moreover, the rate of muscle strength loss (dynapenia) progresses 2–5 times faster than the reduction in muscle mass, suggesting a decline in muscle quality [16,20,21]. Notably, individuals over 75 experience a dramatic decline, losing around 60% of their peak muscle strength and 30% of their physical function [19]. 

However, while the natural aging process can lead to some loss of muscle function, sarcopenia represents a more severe and clinically significant deterioration.

The underlying pathophysiological mechanisms of sarcopenia are multifaceted and complex. With aging, the crucial balance between muscle protein synthesis and breakdown is disrupted, mainly due to a decline in anabolic hormones like testosterone, growth hormone, and insulin-like growth factor 1, which are essential in maintaining muscle mass [5,22]. This hormonal decline is compounded by changes in the metabolic and cellular environments within muscle tissue, such as altered insulin signaling and mitochondrial dysfunction, alongside chronic low-grade inflammation and oxidative stress, all of which impede muscle cell repair and regeneration [23,24,25]. Additionally, the number of muscle satellite cells, vital for muscle repair, diminishes with age, limiting muscle growth and recovery capabilities and thus compromising overall muscle function [22]. 

Neuromuscular degeneration significantly contributes to sarcopenia’s progression. The loss of motoneurons reduces the muscle fiber number and size, while insufficient reinnervation by the remaining neurons diminishes muscle function [26]. Compounded by age-related deterioration at the neuromuscular junction, these changes result in decreased muscle activation and force production [23]. Additionally, sarcopenic muscle exhibits a reduced size and number of myofibers, especially type II fibers, leading to fewer motor units and increased intramuscular fat [23]. 

Moreover, lifestyle factors such as poor nutrition and reduced physical activity not only contribute to the development of sarcopenia but also significantly exacerbate its progression. These factors highlight the role of environmental influences in both triggering and accelerating the decline in muscle mass and strength. Specifically, inactivity exacerbates aging’s effects on muscle, highlighting disuse as a primary factor in the age-related loss of muscle mass and strength [27,28,29,30].

The diagnostic criteria for sarcopenia are generally divided into three primary categories: (a) muscle strength, commonly evaluated using the hand grip strength; (b) muscle mass, typically quantified as the appendicular skeletal muscle mass adjusted for height or body mass index (BMI); and (c) physical performance, frequently assessed using the gait speed [15,31,32,33,34]. Recognizing the established diagnostic cutoffs is crucial in accurately differentiating normal aging from sarcopenia. Among the various frameworks for diagnosis, the European Working Group on Sarcopenia in Older People (EWGSOP2) provides widely recognized criteria [15]. According to the EWGSOP2, sarcopenia is probable when low muscle strength is detected, explicitly defined as hand grip strength of less than 27 kg for men and less than 16 kg for women. For muscle mass, low levels are indicated by an appendicular skeletal muscle mass divided by height squared (ASM/h^2^) of less than 7.0 kg/m^2^ for men and less than 5.5 kg/m^2^ for women. Additionally, a gait speed of less than 0.8 m per second over a short distance may suggest severe sarcopenia [15]. These specific thresholds from the EWGSOP2 illustrate the practical application of the diagnostic criteria and help in the clinical identification and management of sarcopenia.

Sarcopenia is associated with a broad spectrum of adverse health outcomes. This condition not only leads to an increased risk of falls and fractures [35,36] but also has significant implications for functional limitations, making daily activities more challenging and often leading to disability in performing activities of daily living independently [37,38].

Hospitalization and institutionalization become more likely outcomes for individuals suffering from sarcopenia, as their increased care needs and functional limitations may necessitate admission into both short-term and long-term care facilities [39,40]. Furthermore, sarcopenia is an independent predictor of increased mortality risk, highlighting its critical impact on longevity and the urgent need for intervention [41].

Sarcopenia’s influence extends to impaired metabolic health, with a noted association with type 2 diabetes mellitus [42], osteoporosis [43], and cardiometabolic diseases [44], indicating a complex interplay between muscle mass reduction and metabolic dysregulation. This relationship emphasizes the necessity of addressing sarcopenia within the broader context of chronic disease management.

In addition, sarcopenia is strongly associated with various long-term conditions, including musculoskeletal, endocrine, neurological, psychiatric, eye, and cardiovascular conditions [45]. Moreover, sarcopenia is associated with cognitive impairment and depression [46,47], suggesting a link between physical and mental health that warrants further exploration.

This wide range of associations underscores the pervasive impact of sarcopenia on the health and well-being of older adults.

### 3.2. Pelvic Floor Disorders

PFDs refer to a spectrum of clinical conditions resulting from pelvic floor muscle (PFM) and connective tissue dysfunction. These disorders predominantly affect the structural support and function of the pelvic organs, leading to urinary incontinence (UI), anal incontinence (AI), and POP [48].

PFDs represent a significant health concern among adult women, with research indicating their substantial prevalence across various populations. A recent study conducted in Spain involving 1446 women found that approximately 40% of women presented with a single problem, around 17% had two disorders, approximately 6% experienced three problems, and about 2% had four PFDs. UI emerged as the most frequent issue, followed by pelvic pain, symptoms of POP, and FI [49]. In the United States, a study conducted by Wu et al. [50] revealed that approximately one quarter of women encounter at least one form of PFD, with this prevalence notably escalating with age. The rate more than doubles among women aged over 80 years. 

The anticipated trends in the prevalence of PFDs among women in the United States highlight a critical public health concern, reflective of the shifting demographics towards an older female population. Utilizing data from the 2005 National Health and Nutrition Examination Survey and population projections from the U.S. Census Bureau spanning 2010 to 2050, Wu et al. [51] project a substantial rise in the occurrence of PFDs. By 2050, the number of women experiencing at least one PFDs is expected to escalate from 28.1 million in 2010 to 43.8 million. This projection delineates significant increases across specific disorders, with UI predicted to surge by 55%, FI by 59%, and POP by 46%. Such projections are instrumental in underscoring the evolving landscape of women’s health needs directly tied to an aging demographic.

*Urinary incontinence:* UI is defined as “the complaint of any involuntary loss of urine” [8]. This encompasses a range of conditions where the leakage of urine occurs at times that may not be socially or hygienically acceptable and is a significant enough problem to warrant attention. Furthermore, this acknowledges the broad spectrum of experiences among individuals, including those with rare or incidental episodes of UI, highlighting the condition’s varied impact on quality of life and personal distress. UI manifests in several subtypes, each defined by distinct triggers and symptoms.

Stress UI (SUI) occurs when physical activities with abdominal pressure, such as coughing, sneezing, exercising, lifting, or laughing, lead to involuntary urine leakage [52,53]. SUI is attributed to a combination of factors, including weakened PFM, sphincter dysfunction, urethral support loss, and age-related changes in urethral function [54]. However, the two primary pathophysiological mechanisms identified in SUI are urethral hypermobility and intrinsic sphincter deficiency, representing a spectrum rather than distinct entities. Urethral hypermobility is characterized by insufficient pelvic floor support, leading to unequal pressure transmission during intra-abdominal pressure rises. Intrinsic sphincter deficiency involves a failure in the urethral sphincter mechanics, resulting in leakage even with minimal pressure increases [55].

Contrary to SUI, urge UI (UUI) arises from physiological disturbances within bladder function. This condition is marked by a sudden, overpowering urge to urinate that leads to involuntary leakage. UUI is often attributed to detrusor muscle overactivity or neurological factors compromising normal bladder control [56]. Poor detrusor compliance leads to reduced bladder capacity and increased filling pressures, while bladder hypersensitivity, mediated by abnormal sensory signal processing from the bladder, enhances the sensation of urgency [57].

Mixed UI combines SUI and UUI symptoms, presenting a dual challenge of incontinence with an overlap in their pathophysiological mechanisms [53]. Most women do not have pure stress or urge incontinence, and studies show that mixed incontinence is the most common type of urine loss in women [58].

*Anal incontinence:* AI and FI are related but distinct conditions with significant implications for diagnosis and management. FI involves the involuntary loss of fecal material [59], while AI encompasses fecal and gas leakage, impacting social interactions and psychological well-being [60].

The process of defecation is initiated by rectal distension, which leads to rectal contraction, an urgency sensation, and the reflex relaxation of the internal anal sphincter. Socially convenient circumstances allow for PFM relaxation and subsequent defecation, whereas, in other cases, the voluntary contraction of the external sphincter and puborectalis muscles can defer defecation [61]. Within this mechanism, anal continence is maintained through a coordinated effort between the anal sphincters, PFM, rectal and colonic motility, and central and peripheral nervous systems. FI emerges when there are disruptions to this process, which can result from a range of factors, such as disturbances in bowel habits, altered bowel motility, damage or weakening of the anal sphincter muscles, conditions that lead to poor rectal compliance like rectal inflammation, abnormalities in rectal sensation, or dysfunction in the PFM [61,62].

Aging significantly influences FI through various physiological and structural changes in the anorectal region. The internal and external anal sphincters undergo changes such as fibrosis, thinning, and decreased muscle strength, leading to a reduced resting tone and maximum squeeze pressures [63,64]. This sphincter deterioration is a key contributor to FI’s increased occurrence in the elderly. Additionally, the aging process compromises PFM support, introducing laxity that alters the anorectal angle and disrupts the continence mechanism. Age-related declines in rectal sensation and compliance further exacerbate the FI risk by impairing the rectum’s reservoir function. Decreased sensation extends the time to recognize rectal filling, and diminished compliance restricts stool accommodation, heightening the likelihood of incontinence episodes [65,66].

*Pelvic organ prolapse:* POP is defined by the International Continence Society and the International Urogynecological Association as the descent of one or more of the anterior vaginal wall, posterior vaginal wall, the uterus (cervix), or the apex of the vagina (vaginal vault or cuff scar post-hysterectomy). According to this definition, it is essential to correlate the presence of any such anatomical changes with relevant symptoms experienced by the woman, which may include alterations in normal sensation, structure, or functional capabilities in relation to the position of her pelvic organs [53].

The most frequently reported symptoms include sensations of a vaginal lump or bulge and a ‘dragging’ sensation within the pelvis, which can profoundly affect a woman’s quality of life [67]. Symptomatic individuals may describe a sensation akin to sitting on a ball or an egg and may observe tissue protruding through the vagina, complicating routine activities such as voiding or passing stool [68]. The symptom complex of POP extends beyond bulge symptoms to include pelvic pressure, groin pain, low back pain, painful intercourse, difficult bowel movements, UI or FI, and sexual dysfunction, encompassing difficulties in achieving orgasm and diminished vaginal sensation [69,70,71]. Notably, research indicates that a significant proportion of women with POP exhibit signs of central sensitization, reflecting an enhanced response of the central nervous system to stimulation, which may exacerbate pain perception [72].

The pathophysiology of POP is characterized by biomechanical and structural alterations within the pelvic floor and its supporting tissue. The weakening or damage to the PFM, endopelvic fascia, and pelvic ligaments undermines their capacity to sustain the pelvic organs, which leads to their downward displacement into the vaginal canal [73]. This condition is marked by changes in the composition and structure of collagen and elastin in the pelvic tissue, leading to diminished elasticity and structural integrity [74]. Furthermore, the normal distribution of intra-abdominal pressure is disrupted due to compromised support, exacerbating the organ prolapse [73]. Consequently, this disruption results in the mechanical displacement of the organs and potentially impairs their functionality, manifesting as urinary, bowel, and sexual dysfunction [75]. Aging plays a pivotal role, contributing to tissue denervation, devascularization, anatomical changes, and increased collagen degradation, alongside hormonal variations, all of which may reduce mechanical strength and predispose individuals to POP [76].

### 3.3. Bidirectional Relationships between Sarcopenia and PFDs

Current research suggests a bidirectional relationship between sarcopenia and PFDs in the aging population, revealing not just a coincidental co-occurrence but a potential causative interplay that may exacerbate the severity and implications of each disorder.

Sarcopenia’s Impact on PFDs: Several studies have demonstrated how sarcopenia can influence the development and severity of PFDs. Specifically, a longitudinal study found that variations in body composition and muscle strength are closely linked to the risk of SUI and UUI. It revealed that a decline in grip strength of 5% or more significantly increases the likelihood of these disorders, whereas an improvement in appendicular lean mass reduces their occurrence [77]. Further reinforcing this connection, cross-sectional studies have directly linked UI to diminished muscle mass and strength, emphasizing the crucial role of sarcopenia in the development of UI [78,79]. Additional research has indicated that severe forms of POP often coexist with sarcopenia, suggesting that muscle deterioration might exacerbate the severity of POP [12]. This is complemented by findings that deteriorated muscle quality, especially in the psoas muscle, is significantly associated with the increased severity of POP [80]. The relationship between sarcopenia and FI, particularly in patients with dysphagia, has also been investigated, revealing a significant increase in the risk of FI associated with sarcopenia [81]. Furthermore, rehabilitation outcomes for patients suffering from sarcopenia also show that sarcopenia adversely affects the recovery of independence in basic pelvic functions, such as urination and defecation [82]. These findings collectively demonstrate a significant association between sarcopenia and the lifecycle of PFDs. Sarcopenia may contribute to the initiation of these conditions, potentially exacerbate their severity, and complicate recovery efforts through mechanisms such as decreased muscle strength and the reduced force-generating capacity of the PFM. The muscle deterioration associated with sarcopenia, including a decrease in the cross-sectional area of type II muscle fibers and an increase in fibrosis, is associated with an increased risk of incontinence and POP [83,84,85,86]. Detailed mechanisms are discussed in the ‘Mechanisms of Interaction’ section.

PFDs’ Impact on Sarcopenia: Despite the paucity of research exploring the reverse relationship, evidence suggests that PFDs may also lead to sarcopenia. 

Given that significant functional impairments are key indicators in the diagnosis of sarcopenia, research examining the relationship between new-onset urinary incontinence and physical performance measures like gait speed and timed chair stand tests has found that urinary incontinence could serve as an early indicator of this muscle-wasting condition [87]. This link is supported by findings that older adults with fecal incontinence symptoms experienced significant declines in physical performance over time, suggesting a direct connection between PFDs and the development or worsening of sarcopenia [88]. Additionally, a recent survey [89] found that 46% of women with symptomatic pelvic floor disorders ceased participating in specific physical activities, particularly high-impact sports and gym-based strength training, which are crucial in maintaining muscle mass and strength. These behavior changes highlight the impact of PFDs on physical activity levels, potentially initiating or exacerbating sarcopenia. 

Evidence suggests that pelvic floor disorders may influence the progression of sarcopenia, particularly by limiting physical activity and impairing routine daily functions. While studies indicate that the symptoms of PFDs, such as incontinence, could lead to decreased overall physical performance and potentially accelerate sarcopenia, the precise nature of this relationship warrants further detailed investigation to develop effective integrated treatment strategies.

### 3.4. Mechanisms of Interaction

Epidemiological data reveal an overlap in the prevalence of sarcopenia and PFDs among older adults, suggesting shared risk factors and possibly common pathological pathways. Despite this observed co-occurrence, the underlying mechanisms that intertwine these conditions remain underexplored [90]. Recognizing this gap, it is important to examine the shared pathophysiological processes underlying sarcopenia and PFDs, highlighting the potential commonalities in their development.

The pelvic floor is a complex anatomical entity comprising muscles, ligaments, and fascial components, intricately woven to support the pelvic organs. This structure primarily includes the levator ani muscle group, consisting of the puborectalis, pubococcygeus, and iliococcygeus muscles, alongside the urogenital diaphragm. Equally important are the sphincter muscles, including the urethral and anal sphincters, which are essential in maintaining control of urination and defecation [7]. Through proper coordination with the nervous system, these elements collectively play a pivotal role in ensuring continence, supporting organ suspension, and facilitating functions such as voiding, defecation, and sexual activities. In responding to increases in intra-abdominal pressure, such as during coughing, the PFM contract involuntarily to uphold the pelvic organ support, closing the urethra, anus, and vagina to maintain continence while also enabling voluntary control for urination, defecation, and sexual functions through sphincter management and organ positioning [91]. The PFM are critical in maintaining pelvic girdle stability, illustrating a finely tuned balance between structural support and functional capability.

Beyond its local significance, the pelvic floor is not an isolated entity. However, it is integrated with distant body structures through myofascial connections, establishing a network that interacts with the abdominal muscles and diaphragm, extending its influence to the feet and neck [9,92,93]. Integrating the pelvic floor with the abdominal and surrounding musculature forms a functional unit essential in stabilizing the body posture, contributing to respiratory processes, and participating in locomotion, emphasizing the necessity of considering these interrelations when addressing the pelvic floor’s pathophysiology [9].

Research has shown that the PFM undergo progressive age-related muscle deterioration, similar to sarcopenia’s effects on other skeletal muscles. Specifically, a decrease in the cross-sectional area of PFM, associated with aging, correlates with an increased risk of UI [83]. Furthermore, studies on the obturator internus muscle, a key component of the pelvic floor, reveal age-related reductions in its force-generating capacity and an increase in fibrosis, particularly in individuals over 60 years [94]. Additionally, the study by Alperin et al. [84] identifies that aging significantly reduces the physiological cross-sectional area and increases fibrosis in the PFM, suggesting a notable decline in the force-generating capacity of these muscles. Further supporting the link between age-related sarcopenia and PFDs, a study by Yaşar et al. [95] utilized static MRI to demonstrate that the physiological cross-sectional area of the PFM, specifically the puborectalis part of the levator ani, significantly decreases with age in women with stress and mixed UI. Furthermore, the study by Neshatian et al. [80] highlights that the severity of POP is associated with an increasing psoas muscle fat fraction, a biomarker indicative of sarcopenic changes, suggesting that sarcopenia could significantly influence the exacerbation of POP. Additionally, drawing from the anatomical insights provided by the literature, it was postulated that the weakening of the iliococcygeus muscle may precipitate POP, whereas a diminishment in the strength of the puborectalis muscle is implicated in the onset of UI [85]. These insights draw a direct parallel between the aging impact on the PFM and the broader phenomenon of muscle deterioration seen in sarcopenia, emphasizing the importance of addressing age-related musculoskeletal changes in the context of PFDs.

Similar to sarcopenia, characterized by a reduction in muscle fiber size and number, especially in type II (fast-twitch) fibers, which are known for their reliance on anaerobic metabolism and their pivotal role in short bursts of high-intensity activity [23], studies on the PFM also reveal analogous alterations [86]. These fast-twitch fibers within the PFM are essential in maintaining continence in the rapid, forceful contractions that increase the pressure in the urethral and anal sphincters during activities that raise the intra-abdominal pressure, such as coughing, laughing, or physical exertion, thereby preventing urine and fecal leakage [7,96]. The decline in type II fiber functionality underscores a mutual pathway influencing UI and FI, reflecting the systemic nature of the muscle deterioration observed in sarcopenia and its implications for pelvic floor health.

Building on the understanding of sarcopenia’s impact on the muscle fiber size and number, including the significant role of type II fibers, the study by Dias et al. [97] extends this narrative to the neural control properties of the PFM in the context of aging. Their research elucidates that aging is associated with a notable decrease in motor unit firing rates and an increase in motor unit action potential amplitudes within the external anal sphincter, highlighting an age-related decline in neural excitation and muscular responsiveness. These findings mirror the neural and muscular deterioration seen in sarcopenia, suggesting a comprehensive age-related decline across both the skeletal and PFM that contributes to the increased prevalence of FI among the elderly [23,26,97].

### 3.5. Shared Risk Factors

In addition to the physiological changes and neural alterations observed in aging populations, it is crucial to consider the shared risk factors contributing to the development of sarcopenia and PFDs.

Physical inactivity is a recognized precursor to the onset and progression of sarcopenia, influenced by conditions such as disease-related immobility or disability, as well as lifestyle choices that favor minimal physical engagement. Such inactivity is linked to a cascade of adverse effects, including muscle weakness, which further discourages physical activity and decreases muscle quality and strength [29,30]. Furthermore, UI is recognized as a significant deterrent to physical activity, establishing a complex interrelationship between physical inactivity, sarcopenia, and PFDs [98]. The interplay between these conditions suggests a vicious cycle where the muscle weakening associated with sarcopenia adversely affects the pelvic floor’s structural and functional integrity, increasing the susceptibility to PFDs. Meanwhile, the challenges posed by PFDs can lead to a reluctance to engage in physical activities, exacerbating the muscle loss and weakness characteristic of sarcopenia. Evidence from a study on older adults residing in nursing homes underscores the significant interplay between sarcopenia and PFDs, highlighting sedentary behavior as a significant exacerbating factor for both conditions. The findings demonstrate how physical inactivity not only accelerates the decline in muscle mass and strength associated with sarcopenia but also contributes to the weakening of the PFM, leading to an increased risk of PFDs such as UI. Furthermore, the study illustrates that individuals with PFDs may experience discomfort, pain, or a fear of incontinence during physical activities, prompting a reduction in their engagement, creating a vicious cycle that amplifies the severity of sarcopenia and further impairs the pelvic floor functionality [99]. Complementing the evidence on PFDs as a deterrent to physical activity, in a large cohort study involving 2914 older adults, 8% reported symptoms of FI, which was associated with significant declines in physical performance over four years. This research further highlights the significant impact of PFDs on physical performance among older adults. It was found that individuals with FI symptoms exhibited notable declines in physical performance over time, underlining the complex relationship where FI is not only potentially influenced by factors such as physical inactivity and muscle weakness but also contributes to the cycle of decreased physical engagement due to the accompanying social and psychological burdens [88].

Another study focusing on the prevalence of sarcopenia in older women with PFDs highlighted the significant presence of sarcopenia among those with POP, indicating that muscle weakening associated with sarcopenia could exacerbate the severity of POP. This aligns with the understanding that physical inactivity can lead to sarcopenia, further impairing the pelvic floor’s integrity and functionality, thereby heightening the vulnerability to PFDs such as POP [12]. However, groundbreaking research from the UK Biobank presents a counterintuitive perspective, showing that more time spent in light-intensity physical activity was associated with an increased risk of developing POP among middle-aged and elderly women. This prospective cohort study revealed that every additional hour per day spent in light-intensity physical activity elevated the risk of POP by 18%, while an increase in sedentary time was inversely associated with the POP risk [100]. These findings challenge the conventional approach that all forms of physical activity uniformly confer protective effects against PFDs and suggest a more complex relationship that may vary with the intensity of the activity.

Metabolic syndrome emerges as a significant risk factor for both sarcopenia and PFDs, revealing complex interconnections that underscore the systemic nature of these conditions. In sarcopenia, metabolic syndrome contributes to muscle degradation through insulin resistance, reducing muscle protein synthesis and increasing muscle breakdown. This is compounded by the inflammatory state associated with metabolic syndrome, which can further accelerate muscle loss [101]. Concurrently, metabolic syndrome influences the development of PFDs by exacerbating obesity-related pressures on the pelvic floor, altering adipokine profiles, and fostering systemic inflammation that can weaken the PFM and connective tissue [102]. Therefore, the shared pathways of insulin resistance and chronic inflammation establish metabolic syndrome as a pivotal common risk factor that intricately links the pathogenesis of sarcopenia and PFDs.

Nutritional deficiencies, particularly in protein, vitamin D, and minerals, directly affect both skeletal muscle health and the integrity of the pelvic floor, contributing to sarcopenia and PFDs [28,103]. These deficiencies reduce muscle mass and function, setting the stage for sarcopenia [28]. Additionally, evidence shows that these nutritional deficiencies also weaken the PFM, thereby increasing the risk of PFDs [103]. Moreover, constipation represents a critical intersecting point in this relationship, arising from sarcopenia due to the reduced functionality of the abdominal and pelvic muscles and directly from poor nutritional habits [48,104]. The mechanism by which constipation exacerbates PFDs involves increased intra-abdominal pressure during straining efforts to evacuate, which places additional strain on the PFM. Over time, this repetitive stress can further weaken these muscles and supporting structures, leading to or worsening existing PFDs [9]. 

Menopausal hormonal influences: Menopause, which involves a significant decline in estrogen levels, is associated with various changes in musculoskeletal health, potentially influencing both sarcopenia and PFDs. Research shows that estrogen influences muscle protein synthesis and degradation, essential mechanisms for muscle health [105]. It is also suggested that estrogen inhibits disuse-induced muscle atrophy and stimulates regeneration [106]. Estrogen modulates mitochondrial function in the skeletal muscle, impacting energy production and the oxidative capacity, which is important for muscle endurance. It also regulates genes involved in mitochondrial biogenesis and influences satellite cell activity, which is necessary for muscle repair and regeneration [106,107]. Evidence also indicates that estrogen protects the skeletal muscle from apoptosis, helping to defend against muscle mass loss [106].

Studies also suggest that estrogen plays a role in modulating the inflammatory responses that affect muscle health. A decrease in estrogen levels is associated with an increase in pro-inflammatory cytokines such as IL-6 and TNF-α [108,109]. These cytokines can contribute to muscle catabolism, potentially exacerbating muscle mass loss and strength decline in postmenopausal women. Estrogen is observed to mitigate these inflammatory processes, suggesting a role in protecting against inflammation-induced muscle degradation [110]. Furthermore, it influences the ubiquitin–proteasome system involved in protein degradation. Changes in this system during menopause may lead to increased muscle protein breakdown, contributing to the higher prevalence of muscle wasting among postmenopausal women [106].

While the research on estrogen’s role in musculoskeletal health is insightful, it is predominantly derived from in vitro studies. This highlights a significant gap in its clinical translation to human models, particularly in longitudinal studies with postmenopausal women. Thus, further research involving human subjects is essential to fully understand and verify these mechanistic findings and their implications for menopausal healthcare.

Specifically regarding PFDs, the presence of abundant estrogen receptors in the pelvic floor indicates that estrogen depletion could significantly impact these structures [77,111]. However, whether these effects are comparable to those observed in the broader musculoskeletal system is uncertain, underscoring the need for cautious interpretation. The hormonal depletion is linked to increased vulnerability to PFDs, such as UI and POP [112]. Estrogen deficiency affects collagen production, tissue elasticity, and neurovascular support, weakening the pelvic tissue and increasing the susceptibility to prolapse and incontinence [77,113,114].

Although some research has explored estrogen’s role in skeletal muscle health, studies on its effects on the pelvic floor during menopause are still scarce. Findings suggest a potential relationship between estrogen deficiency and the onset of PFD symptoms, including both structural failures like prolapse and functional disturbances such as incontinence. However, the specific mechanisms remain under-researched and poorly understood, highlighting the need for further investigation [115,116]. 

Considering the possible roles that sex hormones play in both sarcopenia and PFDs, hormone therapy (HT) emerges as a potential intervention. However, the application of estrogen in managing these conditions is nuanced. Several studies have evaluated the effect of estrogen replacement therapy on sarcopenia parameters in postmenopausal women and have reported a positive effect on muscle strength and function [117,118]. Despite these findings, for sarcopenia in postmenopausal women, estrogen is not a recommended treatment due to its unconfirmed efficacy and the potential for severe adverse effects [119,120]. 

Similarly, the application of estrogen therapy in treating PFDs in postmenopausal women presents both potential benefits and challenges. Local estrogen treatments have demonstrated effectiveness in alleviating symptoms of UI by enhancing tissue quality and function [121], but not for FI [122] and POP [123]. Evidence for the effectiveness of systemic hormone therapy in these conditions is inconclusive and sometimes contradictory. Systemic hormone therapy has been associated with an increased risk of developing or worsening urinary incontinence and does not show consistent benefits in terms of preventing or treating PFDs [115]. 

Given these mixed outcomes, the use of hormone therapy for PFDs requires the careful consideration of individual patient factors and potential side effects, emphasizing the need for tailored therapeutic approaches based on the most recent research findings.

Exploring shared risk factors underscores the complex interplay between lifestyle, metabolic health, and nutrition in the pathogenesis of sarcopenia and pelvic floor PFDs. These insights lay the groundwork for holistically advanced patient care strategies that address these multifaceted concerns, highlighting the importance of integrated approaches in managing these age-related conditions.

To encapsulate the critical points discussed in this section, we present Table 1 below, which outlines the main findings regarding the bidirectional relationships, mechanisms of interaction, and shared risk factors between sarcopenia and PFDs.

### 3.6. Implications for Patient Care

Integrated Screening Approach: This review underscores the significance of combined screening for sarcopenia and PFDs as they have well-documented physiological and functional interconnectedness. Specifically, it promotes an insightful assessment recognizing the complex link between the two phenomena. This approach does not suggest creating novel screening tools but utilizing the existing validated instruments for each pathology in collaboration. The innovative aspect lies in the systematic integration of assessments for both conditions within routine clinical evaluations, thus enabling early identification and management.

Extend Screening to Underlying Conditions: A critical outcome of this analysis underlines the need to extend the screening scope to include underlying conditions that contribute to both sarcopenia and PFDs. These conditions, such as metabolic imbalances and nutritional deficiencies, are known to play a critical role in their development [28,101,102,103]. We believe that a holistic screening approach that includes evaluating these contributory factors is essential to improve the quality of care for both conditions.

Addressing Cyclical Contributing Factors: Our review highlights the importance of recognizing and addressing the cyclical contributing factors, where one condition may trigger the other—for instance, the way in which sarcopenia can lead to an increased risk of PFDs through mechanisms like constipation or reduced mobility, and, in contrast, how PFDs may contribute to sarcopenia by limiting physical activity. Identifying these cycles offers a substantial opportunity for preventative care, allowing for interventions that treat the existing conditions and disrupt the pathological feedback loops.

Adapting Resistance Exercise for Patients with PFDs: Resistance exercises are a primary intervention for the prevention and treatment of sarcopenia, allowing one to preserve or regain physical capabilities [27]. The dose–response relationship between PA and sarcopenia has been well established, demonstrating that regular PA, particularly resistance training in a progressive manner, is crucial in preserving or increasing muscle mass and strength, thereby mitigating the effects of sarcopenia [124]. This makes physical activity the most significant modifiable driver of sarcopenia [125]. However, the relationship between PA and PFDs is less clear. While PA generally benefits overall health and can improve PFM function through indirect mechanisms, such as weight management and enhanced general fitness, the specific dose–response relationship between PA and PFDs is not established [126]. 

Current evidence suggests that mild to moderate PA may decrease the risk of UI and other PFDs, but the impact of high-intensity or light-intensity PA remains controversial and varies depending on the type, intensity, and duration of the exercise, as well as individual patient factors [101,127]. One possible reason for the lack of a well-defined dose–response relationship between PA and PFDs is the complexity and variability of PFDs themselves. PFDs encompass a range of conditions, each with different pathophysiological mechanisms and risk factors [126]. While these conditions may present separately, they can also influence one another; for instance, POP can lead to UI [128]. Additionally, the impact of PA on the pelvic floor can vary significantly based on individual patient characteristics such as age, parity, hormonal status, baseline pelvic floor muscle strength, and previous physical activity habits [129]. Furthermore, methodological differences in studies, including how PA and PFDs are measured and reported, contribute to their inconsistent findings. The heterogeneity of the study populations and the lack of standardized protocols for the assessment of PA and PFD outcomes further complicate the establishment of a clear dose–response relationship [127].

Despite these challenges, evidence suggests a relationship between exercise intensity and PFM strength. For example, the maximum PFM voluntary contraction (MVC) is notably lower in women who engage in strenuous or high-impact exercise compared to those who participate in mild or moderate activities [130]. This finding underscores the importance of tailoring exercise regimens to enhance the pelvic floor muscle functionality, particularly advocating for moderate-intensity exercises that support rather than strain these muscles [130]. Moreover, women engaging in moderate exercise exhibit better control and strength in the pelvic floor, leading to favorable outcomes in managing PFD symptoms. For instance, to minimize stress on the pelvic floor, focusing on supine moderate-intensity exercises may be advisable, while avoiding prolonged periods of light-intensity physical activity [100]. 

Given the conflicting literature on the impact of the exercise intensity on PFDs, exercise regimens for sarcopenic patients must be carefully tailored to ensure that they do not exacerbate PF symptoms. Additionally, a systematic review emphasizes that exercises lacking targeted PFM contraction are not effective treatment regimens for PFDs such as urinary incontinence [131]. To effectively address this, it is essential to incorporate specific PFM exercises into the recommended approach to improve the morphology and muscle function of the pelvic floor. Thus, given the special considerations for sarcopenia patients, integrating PFM exercises with resistance training is essential. Research indicates that voluntary PFM contractions during exercise can help to prevent negative impacts on the pelvic floor, such as bladder neck descent and hiatal area enlargement, which are risks associated with high-impact or strenuous activities [130].

For optimal outcomes, incorporating low-impact strength-training modalities like Pilates, along with targeted pelvic floor exercises, ensures the active engagement of these muscles, enhancing both pelvic floor function and overall muscle strength [132]. Research highlights that supervised and intensive pelvic floor muscle exercises are significantly more effective than unsupervised exercises. Such structured and closely monitored regimens not only improve the symptoms of SUI and POP but are also associated with no known adverse effects, making them a safe and essential component of pelvic health management [133]. This balanced approach not only addresses the muscle deterioration common in sarcopenia but also supports pelvic health, preventing complications like the exacerbation of PFDs.

Multidisciplinary Approach: Our review underlines the necessity of a multidisciplinary strategy in managing sarcopenia and PFDs, integrating the expertise of geriatricians, urogynecologists, physical therapists, dietitians, and mental health professionals. This team approach could provide comprehensive care for both conditions and their bidirectional effects, resulting in improved patient outcomes.

To further illustrate these key practical implications, we summarize them in Table 2 below.

### 3.7. Implications for Research

Exploration of Integrated Physical Therapy Methods: Despite the recognized benefits of resistance exercise for sarcopenia [124], the application of targeted physical therapy techniques such as biofeedback, electrostimulation, and the use of vaginal cones remains unexplored in patients with concurrent PFDs. Future research should evaluate the effectiveness and safety of incorporating these techniques in patients with sarcopenia and PFD, without worsening their PF symptoms.

Longitudinal Studies on the Bidirectional Relationship: There is a significant gap in longitudinal studies on the progression of sarcopenia and PFDs over time in the same cohort. Examining how these conditions influence each other over the long term could provide invaluable insights into their interplay and guide the development of proactive, preventive strategies.

Evaluate the Efficacy of Multidisciplinary Care Models: This review suggests a shift towards comprehensive, interdisciplinary care approaches. Future studies should evaluate the efficacy of such models in managing sarcopenia and PFDs, particularly looking at outcomes related to patient satisfaction, quality of life, and healthcare utilization.

## 4. Conclusions

The bidirectional relationship between sarcopenia and PFDs gives more insight into the complex interconnection between these two clinical conditions. Current evidence suggests that sarcopenia and PFDs not only coexist but may also exacerbate each other’s severity and progression. Therefore, an integrated screening and management approach is essential. Furthermore, similar risk factors, particularly physical inactivity, metabolic syndrome, or nutritional deficiencies, result in the development and exacerbation of both conditions. Considering the bidirectional relationship and shared risk factors, healthcare professionals may need to adopt a more integrated approach to increase the quality of care. Moreover, longitudinal research is required to study the natural course of the development of sarcopenia and PFDs and examine the applicability of multidisciplinary models during their management. Finally, the further integration of physical therapy interventions, including tailor-made exercise plans, may be required to prevent the exacerbation of the symptoms.

In conclusion, the evidence above illustrates the need for a paradigm shift towards an integrative approach and preventive care model that prioritizes patient-oriented and interdisciplinary work. By addressing the nature of sarcopenia and PFDs, healthcare professionals can significantly improve the management of these conditions, enhancing the quality of life of the aging population. 

## 5. Limitations

This review is based on existing studies and literature with inherent methodological biases or limitations. The synthesis of these studies may not capture all aspects of the bidirectional relationship between sarcopenia and PFDs. Additionally, the heterogeneity of the study populations and differing diagnostic criteria for sarcopenia and PFDs across studies may limit the generalizability of the findings. As a narrative review, this study does not employ a systematic literature search and selection approach, potentially introducing selection bias. Further research is needed to confirm these findings and explore the mechanisms underlying the relationship between sarcopenia and PFDs in diverse populations.

## Figures and Tables

**Table 1:** Summary of main findings on the bidirectional relationship between sarcopenia and pelvic floor disorders.

Category	Main Findings	Details
Bidirectional Relationship between Sarcopenia and PFDs	Sarcopenia’s Impact on PFDs	Sarcopenia contributes to the initiation of PFDs, exacerbates their severity, and complicates recovery efforts through mechanisms such as decreased muscle strength and reduced force-generating capacity in the PFM. Reduced muscle mass and quality increase the risk of SUI, UUI, and POP. Sarcopenia-associated muscle deterioration, including a decreased cross-sectional area and increased fibrosis of type II muscle fibers, are critical factors in these conditions.
	PFDs’ Impact on Sarcopenia	PFDs lead to reduced physical activity due to discomfort and a fear of incontinence, which accelerates sarcopenia progression. UI and FI contribute to declines in physical performance and muscle strength. The reduction in physical activity associated with PFDs inhibits the maintenance of muscle mass and quality, further exacerbating sarcopenia.
Mechanisms of Interaction	Muscle Deterioration	The age-related deterioration of the levator ani muscle group and sphincter muscles parallels sarcopenia. The decrease in the muscle cross-sectional area and increased fibrosis affect both the pelvic floor integrity and overall muscle function.
	Muscle Fiber Changes	A reduction in type II muscle fibers in PFM and skeletal muscles is a common pathway contributing to PFDs.
	Neural Control	Decreased motor unit firing rates and increased motor unit action potential amplitudes in PFM mirror the changes seen in sarcopenia, contributing to PFDs.
Shared Risk Factors	Physical Inactivity	Physical inactivity leads to the deterioration of skeletal and PFM, contributing to a cyclical relationship between sarcopenia and PFDs. This cycle results in an increased risk of both conditions, which further discourages physical activity, exacerbating the progression of sarcopenia and PFDs.
	Nutritional Deficiencies	Deficiencies in protein, vitamin D, and minerals directly affect both skeletal muscle and pelvic floor integrity.
	Metabolic Syndrome	Metabolic syndrome accelerates muscle degradation through insulin resistance and chronic inflammation, contributing to both sarcopenia and PFDs. Obesity-related pressures and altered adipokine profiles further weaken the PFM and connective tissues, increasing the risk of these conditions.
	Menopausal Hormonal Changes	There are indications that estrogen deficiency during menopause may affect muscle protein synthesis, mitochondrial function, and inflammatory responses, contributing to sarcopenia. Estrogen depletion may weaken the pelvic tissue, increasing the susceptibility to PFDs such as UI and POP. However, much of the research is based on in vitro studies, highlighting the need for further clinical research to confirm these findings.

**Table 2:** Summary of practical implications for management of sarcopenia and pelvic floor disorders.

Implication	Description
Integrated Screening and Management	Implement integrated screening protocols in clinical practice to concurrently address sarcopenia and PFDs, recognizing their bidirectional and cyclical relationships and any underlying conditions.
Comprehensive, Interdisciplinary Care	Foster interdisciplinary collaboration among geriatricians, urogynecologists, physical therapists, nutritionists, and psychologists to provide holistic and comprehensive care plans that enhance patient outcomes by addressing the interconnectedness of skeletal muscle integrity, PFDs, and overall health.
Tailored Rehabilitation Programs	Develop personalized rehabilitation programs that specifically address the unique needs of patients with sarcopenia and PFDs, ensuring comprehensive care that covers all aspects of both conditions.
Resistance Training	Incorporate resistance training into rehabilitation programs to maintain muscle mass, muscle strength, and pelvic floor integrity. This includes specific safe and effective exercises for both conditions, such as those that enhance overall muscle strength while also targeting the pelvic floor muscles. Recommendations include progressive resistance training and the inclusion of pelvic floor muscle exercises to improve outcomes without exacerbating PFD symptoms.
